# Fourier transform-based method for quantifying the three-dimensional orientation distribution of fibrous units

**DOI:** 10.1038/s41598-024-51550-5

**Published:** 2024-01-23

**Authors:** Riccardo Alberini, Andrea Spagnoli, Mohammad Javad Sadeghinia, Bjørn Skallerud, Michele Terzano, Gerhard A. Holzapfel

**Affiliations:** 1 Department of Engineering and Architecture, University of Parma, Parma, Italy; 2 Department of Structural Engineering, Norwegian University of Science and Technology (NTNU), Trondheim, Norway; 3 Institute of Biomechanics, Graz University of Technology, Graz, Austria

**Keywords:** Biomedical engineering, Computational methods, Characterization and analytical techniques

## Abstract

Several materials and tissues are characterized by a microstructure composed of fibrous units embedded in a ground matrix. In this paper, a novel three-dimensional (3D) Fourier transform-based method for quantifying the distribution of fiber orientations is presented. The method allows for an accurate identification of individual fiber families, their in-plane and out-of-plane dispersion, and showed fast computation times. We validated the method using artificially generated 3D images, in terms of fiber dispersion by considering the error between the standard deviation of the reconstructed and the prescribed distributions of the artificial fibers. In addition, we considered the measured mean orientation angles of the fibers and validated the robustness using a measure of fiber density. Finally, the method is employed to reconstruct a full 3D view of the distribution of collagen fiber orientations based on in vitro second harmonic generation microscopy of collagen fibers in human and mouse skin. The dispersion parameters of the reconstructed fiber network can be used to inform mechanical models of soft fiber-reinforced materials and biological tissues that account for non-symmetrical fiber dispersion.

## Introduction

Biphasic solids, characterized by a microstructure of fibrous units embedded in a ground substance, are common in both engineered materials and biological tissues. For example, fiber-reinforced materials have emerged as versatile and indispensable components in a wide range of structural engineering applications, ranging from aerospace and automotive to construction, including fiber-reinforced polymers^1–4^, fiber-reinforced concrete^5,6^ and non-woven fabrics^7^. Their importance goes beyond traditional applications and finds increasing application in the medical sciences and for smart materials^8–10^.

In addition, researchers have thoroughly investigated the mechanical behavior of biological tissues with a fibrous microstructure, including cornea, cartilage, skin, and blood vessels. Collagen is of particular interest, it is also the most abundant protein in the human body and plays a role in providing structural integrity and load-bearing functions of tissues. Understanding the distribution and organization of collagen fibers has significant implications for the preliminary diagnosis of pathological situations. For example, alterations in collagen organization have been associated with diseases such as cancer^11–13^, abdominal aortic aneurysms^14–16^, mitral valve disease^17^, keratoconus^18–21^, and genetic disorders such as Marfan syndrome^22^. Quantification of the underlying microstructure of soft tissues is also of paramount importance for constitutive models developed for fibrous biological tissues. Accurate models rely on microstructural parameters, such as the mean fiber orientation and dispersion, to accurately reproduce the nonlinear anisotropic response of tissues under complex loading scenarios^23–26^.

Precise imaging techniques play a crucial role in assessing the architecture of collagen fibers. Several methods, including histological staining^27,28^, polarized light microscopy^29,30^, optical coherence microscopy^31,32^, and second harmonic generation (SHG) microscopy^33–35^ are used to visualize collagen fibers. Among these techniques, SHG microscopy has gained prominence due to its ability to provide three-dimensional, high-resolution images of collagen fibers that can be detected at depth without the need for staining. However, the quantitative three-dimensional (3D) assessment of the fiber orientation distribution (FOD) remains a challenge.

The importance of automated techniques to measure fiber distribution cannot be overlooked. These methods have the potential to revolutionize the analysis of collagen organization by reducing human error, increasing efficiency and enabling the measurement of a greater number of features. Current approaches to quantitatively measure fiber distribution include texture analysis^36,37^, fiber segmentation and tracking algorithms (e.g., the CT-FIRE algorithm)^38,39^, pixel-by-pixel methods using gradients^40,41^ or directional variance^42,43^, Gabor filter methods^28^, and Fourier transform methods^32,44–46^. In particular, Fourier transform-based methods, including wavelet transforms, have shown promise in terms of computational efficiency irrespective of image complexity, computation speed, and reduced sensitivity to noise and fiber crimping^32^.

While numerous methods exist for two-dimensional (2D) measurements of fiber distribution, the transition to 3D analyses raises open technical questions. For example, fibers with a large elevation with respect to the image plane are not captured because their cross-section is quasi-circular and the direction cannot be recognized by the 2D Fourier transform^47^. Also, existing 2D algorithms either focus exclusively on in-plane or out-of-plane measurements, which limits the comprehensive understanding of fiber dispersion. Even if both the in-plane and out-of-plane fiber distributions are measured, the architecture cannot be combined to produce a 3D distribution since it is not possible to reconstruct the 3D distribution from the distribution about two perpendicular directions without information about their covariance.

Few algorithms are explicitly designed for full 3D measurements, leaving a significant gap in the field^48^. Liu et al.^13^ used a pixel-by-pixel method to extract a spatial description of collagen fibers, but then adopted 3D directional variance, an averaged metric to assess fiber concentration levels, and they provided separate in-plane and out-of-plane fiber distributions. Lau et al.^47^ used a 3D Fourier transform method, but the fiber distribution is computed by dividing the 3D image into regions of interests (ROIs) and finding the overall fiber orientation of each region. The fiber distribution within each region is not considered, so information is lost when a region contains two or more fiber families. In addition, the number of measurements is limited to the number of regions of interest.

In this work, we present a novel 3D Fourier transform-based method to characterize the FOD. Our approach provides a complete 3D description of fiber dispersion within an image, facilitates the identification of individual fiber families, and enables quantitative analysis using fitted bivariate von Mises probability density function (PDF) parameters. The algorithm has fast computation times even with large-sized images and is therefore extremely practical for research and clinical applications. To validate the algorithm, 3D images containing artificial fibers are generated to assess its robustness and precision in computing in-plane and out-of-plane distribution parameters. The performance of the algorithm is then verified for mouse and human skin collagen fibers obtained from SHG images.

## Results

### Discrete fiber orientation distribution

**Figure 1:**
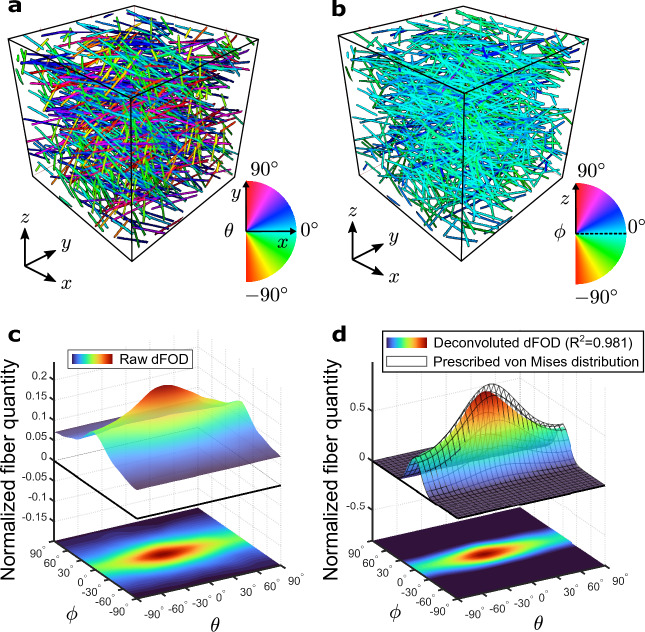
Application of the 3D discrete Fourier transform algorithm: (**a**,**b**) maps of the azimuthal and elevation angles $(\theta ,\phi )$ of a representative artificial fiber dispersion with $N=1000$ fibers of $t=3$ voxel diameter. The prescribed von Mises distribution parameters are $\alpha =\beta =\gamma =0^\circ$, $a=0.5$, $b=5$ ($n=1$); (**c**) corresponding raw dFOD (discrete fiber orientation distribution) $d(\theta ,\phi )$ discretized with $D=31$ angular intervals ($5.8^{\circ }$ angular resolution) and spectrum parameter $q=2.4$; (**d**) deconvoluted dFOD $d'(\theta ,\phi )$, with overlapping prescribed von Mises distribution $\hspace{0.83328pt}\overline{\hspace{-0.83328pt}\rho \hspace{-0.83328pt}}\hspace{0.83328pt}$ (wire-frame plot).

Fibers in biphasic materials are usually dispersed with a non-uniform orientation distribution characterized by one or more fiber families, each having a preferential direction and generally a non-symmetrical dispersion with different in-plane and out-of-plane concentrations. For example, biological tissues such as skin or arterial walls exhibit two distinct fiber families, together with an out-of-plane concentration that is more pronounced compared to the in-plane concentration^49^. In contrast, in 3D printed fiber-reinforced hydrogels, the fibers are dispersed axially along a single preferential direction, while the concentration mainly depends on the diameter of the deposition nozzle and the printing speed^3,9^.

The three-dimensional fiber organization can be described by a FOD function that returns the normalized amount of fibers along all directions in the angular domain of a hemisphere, where $\theta$ and $\phi$ are the azimuthal and elevation angles with respect to the in-plane and out-of-plane directions of a single fiber. The FOD reveals the architecture of the fiber network: the location of a local maximum in the FOD identifies the mean fiber direction, while the standard deviations $\sigma _{\theta \theta }$ and $\sigma _{\phi \phi }$ provide a measure of the in-plane and out-of-plane concentrations. A lower standard deviation corresponds to a higher fiber alignment along the mean direction.

We propose an algorithm that takes advantage of the directional information carried by the 3D discrete Fourier transform to obtain a discrete fiber orientation distribution (dFOD). First, the spectrum of a three-dimensional grayscale image of the fiber network is filtered with a set of filters to obtain a raw dFOD $d(\theta ,\phi )$ (Materials and Methods, Eq. (6)). Then, we remove the interference due to the reciprocal overlaps of the filters in the frequency domain with a deconvolution step to obtain the actual (deconvoluted) dFOD $d'(\theta ,\phi )$. Finally, we estimate the parameters of the fiber orientation distribution by fitting a bivariate von Mises PDF to the deconvoluted dFOD (Materials and Methods, Eqs. (2) and (3)). For multiple fiber families, the parameters of each *n*-th family are obtained by fitting a combination of bivariate von Mises PDFs $\hspace{0.83328pt}\overline{\hspace{-0.83328pt}\rho \hspace{-0.83328pt}}\hspace{0.83328pt}=\sum _n \nu _{\textrm{f},n}\hspace{0.83328pt}\overline{\hspace{-0.83328pt}\rho \hspace{-0.83328pt}}\hspace{0.83328pt}_n$, where $\nu _{\textrm{f,}n}$ is the volume fraction of the family. The parameters of the distribution include the azimuthal and elevation angles $\alpha _n$ and $\beta _n$ of the mean fiber direction, the rolling angle $\gamma _n$ of the family about the mean direction, and the in-plane and out-of-plane concentration parameters $a_n$, $b_n$ (see Fig. 9 in Materials and Methods).

Figure 1 illustrates an application of the algorithm to an artificially generated fiber network. A fiber dispersion image ($256\times 256\times 256$ voxels) with $N=1\,000$ randomly generated fibers of diameter $t=3$ voxels was generated according to a prescribed von Mises distribution ($n=1$) with mean orientation angles $\alpha =\beta =\gamma =0^\circ$ and concentrations $a=0.5$, $b=5$. The raw and deconvoluted dFODs are shown in Fig. 1c,d. In particular, the latter show that the algorithm is able to precisely recover the prescribed von Mises distribution $\hspace{0.83328pt}\overline{\hspace{-0.83328pt}\rho \hspace{-0.83328pt}}\hspace{0.83328pt}$ ($R^2=0.981$).

### Algorithm calibration and validation

We employed artificial fiber stacks with prescribed distributions to calibrate the algorithm and assess its robustness and precision (see Materials and Methods).

#### Calibration

The shape of the deconvoluted dFOD $d'(\theta ,\phi )$ is controlled by a power parameter *q* applied to the spectrum of the fiber image before the filtering process (Eq. (6)). Since the spectrum power parameter affects the standard deviations of $d'(\theta ,\phi )$, we analyzed the relative errors $\Delta \sigma _{\theta \theta }$ and $\Delta \sigma _{\phi \phi }$ between the standard deviations of the prescribed and the measured distributions. For this purpose, we generated four different single-fiber family distributions representing four scenarios of high/low in-plane and out-of-plane concentrations: Case 1: low concentration in both planes ($a=0.5$, $b=0.5$); Case 2: high out-of-plane concentration ($a=0.5$, $b=5$); Case 3: high in-plane concentration ($a=5$, $b=0.5$); Case 4: high concentration in both planes ($a=5$, $b=5$). In total we assumed $\alpha =\beta =\gamma =0^\circ$ and generated 10 three-dimensional images ($256\times 256\times 256$ voxels) each with $N=6\,000$ fibers of diameter $t=3$ voxels.

**Figure 2:**
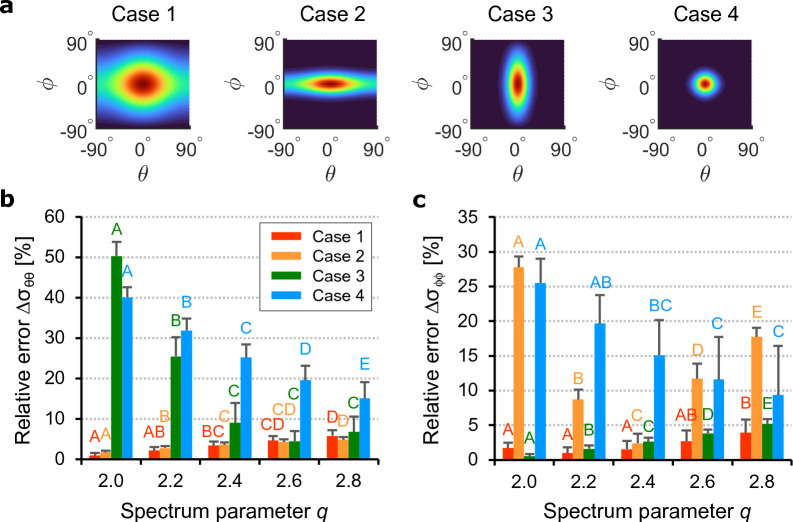
Algorithm calibration: (**a**) contour plots of the prescribed bivariate von Mises distributions ($n=1$, $\alpha =\beta =\gamma =0^\circ$). Case 1: $a=0.5$, $b=0.5$; Case 2: $a=0.5$, $b=5$; Case 3 $a=5$, $b=0.5$; Case 4 $a=5$, $b=5$. Relative errors between the standard deviation of the measured dFOD (discrete fiber orientation distribution) and the prescribed distribution (mean and standard deviation of 10 images); (**b**) error $\Delta \sigma _{\theta \theta }$ along the azimuthal direction $\theta$ for different power parameters *q*; (**c**) error $\Delta \sigma _{\phi \phi }$ along the elevation direction $\phi$ for different *q*. Means not sharing uppercase letters differ significantly by the Tukey-test at the $5\%$ significance level. Letters must be compared among the same cases.

Figure 2a shows contour plots of the prescribed von Mises PDFs. The relative error $\Delta \sigma _{\theta \theta }$ is represented in Fig. 2b. For both Case 1 and 2, the error seems small and increases slightly with *q*, always staying below $6\%$. In Case 3, on the other hand, the error drops quickly from $50\%$ to less than $10\%$ without significant variations between $q=2.4$ and $q=2.8$. In Case 4, the error decreases significantly with increasing values of the spectrum power parameter *q* and reaches its minimum at $q=2.8$. Regarding the relative error $\Delta \sigma _{\phi \phi }$ in the elevation angle, the results are shown in Fig. 2c. In Cases 1 and 3, the error increases with *q* and always stays below $5\%$. In Case 2, the errors are quite high, except for $q=2.4$, as in Case 4, where we observed a decreasing trend for *q*. We also found that errors are higher when concentrations are high along each direction. This is because the standard deviation is smaller at high concentrations and the relative error is more sensitive to small differences in the measured standard deviation since the standard deviation of the prescribed distribution tends to zero.

#### Robustness

We tested the ability of the algorithm to accurately measure dFOD, independently of the fiber number *N* and diameter *t* in the 3D image. The results are also compared in terms of fiber density $\delta$, defined as the average of the voxel intensity over the entire 3D image volume, where $0\%$ corresponds to an empty volume and $100\%$ to completely bright voxels.

**Figure 3:**
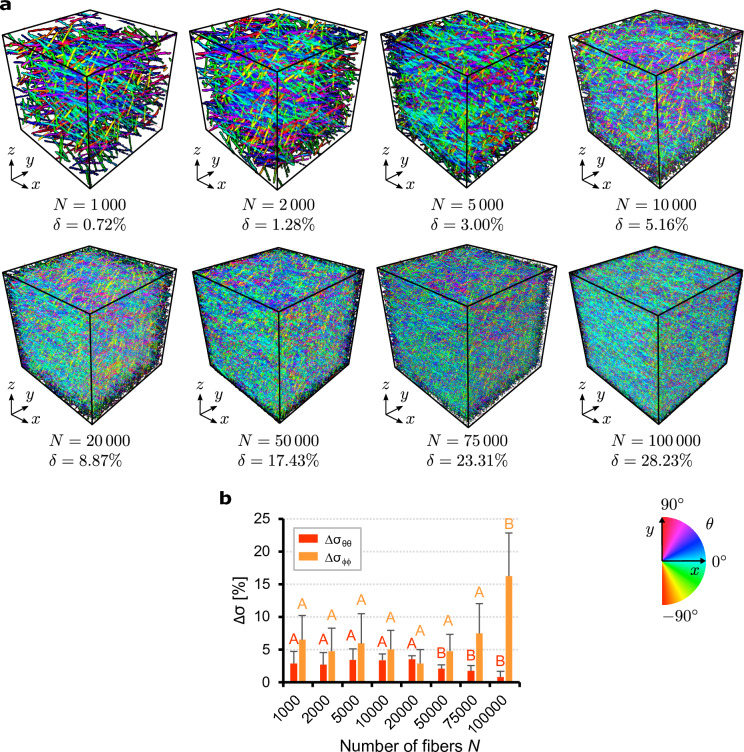
Influence of fiber number: (**a**) maps of the azimuthal angle $\theta$ of 8 representative artificial fiber stacks ($256\times 256\times 256$ voxels), with $N=1\,000$, $2\,000$, $5\,000$, $10\,000$, $20\,000$, $50\,000$, $75\,000$, $100\,000$ fibers of diameter $t=3$ voxels, and distribution parameters $a=0.5$, $b=5$, $\alpha =\beta =\gamma =0^\circ$; (**b**) relative errors $\Delta \sigma _{\theta \theta }$ and $\Delta \sigma _{\phi \phi }$ between the standard deviations of the measured dFOD (discrete fiber orientation distribution) and the prescribed distribution (mean and standard deviation of 10 images). Means that do not use capital letters differ significantly by the Tukey-test at the $5\%$ significance level. Letters must be compared in the same cases.

We generated three-dimensional images ($256\times 256\times 256$ voxels) with increasing numbers of fibers and same diameter $t=3$ voxels. Representative distributions with parameters $a=0.5$, $b=5$, $\alpha =\beta =\gamma =0^\circ$ ($n=1$) were generated containing $N=1\,000$, $2\,000$, $5\,000$, $10\,000$, $20\,000$, $50\,000$, $75\,000$, $100\,000$ fibers, corresponding to fiber densities between $\delta =0.72\%$ and $\delta =28.23\%$ (Fig. 3a). For each case 10 three-dimensional images are generated.

As above, we assessed the algorithm performance by computing the relative errors in the standard deviations along the azimuthal and elevation directions (Fig. 3b). It was found that the error $\Delta \sigma _{\theta \theta }$ depends slightly on the fiber number *N* and generally reduces at higher *N*. This trend might change at higher *N* because for $N \rightarrow \infty$ ($\delta \rightarrow 100\%$) fibers cannot be distinguished and no distribution can be measured. However, a volume with $N = 100\,000$ fibers ($\delta =28.23\%$) represents a density beyond that observed in real collagen fiber tomographies, as shown in the following applications to skin tissue ($\delta = 16.97\%$ for human skin, $\delta = 16.31\%$ for mouse skin). With regard to the elevation angle, the error $\Delta \sigma _{\phi \phi }$ appears to be practically insensitive to *N* and shows small values, except for $N = 100\,000$.

**Figure 4:**
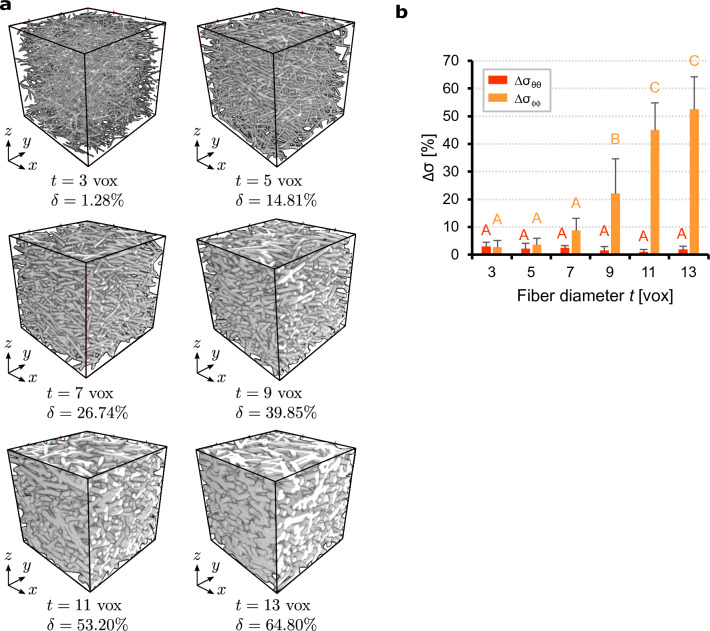
Influence of fiber diameter: (**a**) representative artificial fiber stacks ($256\times 256\times 256$ voxels), generated with $N=2\,000$ fibers and diameters of 3, 5, 7, 9, 11 and 13 voxels. Distribution parameters: $a=0.5$, $b=5$, $\alpha =\beta =\gamma =0^\circ$; (**b**) relative errors $\Delta \sigma _{\theta \theta }$ and $\Delta \sigma _{\phi \phi }$ between the standard deviations of the measured dFOD (discrete fiber orientation distribution) and the prescribed distribution (mean and standard deviation of 10 images). Means not sharing uppercase letters differ significantly by the Tukey-test at the $5\%$ significance level. Letters must be compared among the same cases.

To assess the performance of the algorithm in terms of the fibers diameter, we analyzed artificial fiber stacks with the same number of fibers $N=2\,000$ and increasing fiber diameters of $t=3$, 5, 7, 9, 11 and 13 voxels, corresponding to densities from $\delta =1.28\%$ to $64.80\%$. For each diameter we generated 10 three-dimensional images ($256\times 256\times 256$ voxels) with same dispersion parameters, $a=0.5$, $b=5$, $\alpha =\beta =\gamma =0^\circ$ ($n=1$). Representative images for each diameter are shown in Fig. 4a. As illustrated in Fig. 4b, the algorithm appears to be precise and insensitive to the fiber diameter along the azimuthal direction and predicts the true standard deviation with an error $\Delta \sigma _{\theta \theta }$ smaller than $3.5\%$, with no significant variations. On the other hand, the error along the elevation direction is small only for fiber diameters between 3 and 7 voxels ($\Delta \sigma _{\phi \phi }<8.7\%$) and increases thereafter to about $50\%$ for $t=13$ voxels.

Interestingly, the behavior observed in these two analyses is similar and is directly related to fiber density. In fact, by comparing the errors in Figs.3b and 4b with respect to the density $\delta$, it follows that $\Delta \sigma _{\theta \theta }$ and $\Delta \sigma _{\phi \phi }$ are correlated. The errors $\Delta \sigma _{\theta \theta }$ are small in in both cases and almost insensitive to the fiber density, while $\Delta \sigma _{\phi \phi }$ is small only for densities lower than $\delta \approx 25\%$ ($N=75\,000$, $t=3$ voxels; $N=2000$, $t=7$ voxels) and increases significantly for densities $\delta >28\%$ ($N=100\,000$, $t=3$ voxels; $N=2000$, $t=9$ voxels). This suggests that the algorithm only responds to fiber density and not individually to the number of fibers or their diameter. However, the difference between $\Delta \sigma _{\theta \theta }$ and $\Delta \sigma _{\phi \phi }$ is probably due to the small prescribed standard deviation $\sigma _{\phi \phi }$ (higher concentration) along the elevation direction, increasing the relative error even though the absolute error is small.

#### Precision

The precision of the algorithm is measured by its ability to estimate the parameters of the in-plane and out-of-plane distributions. For each of the combinations of parameters described below, a series of 10 three-dimensional images ($256\times 256\times 256$ voxels) was generated with $N=6\,000$ fibers of $t=3$ voxel diameter.

**Figure 5:**
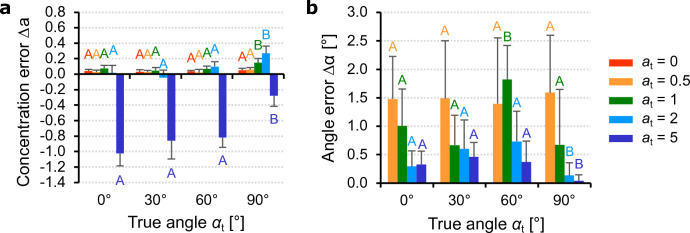
Algorithm precision for in-plane parameters. Errors between the estimated (subscript $\textrm{e}$) and true (subscript $\textrm{t}$) in-plane parameters (mean and standard deviation of 10 images): (**a**) error in the in-plane concentration $\Delta a=a_{\textrm{e}}-a_{\textrm{t}}$; (**b**) error in the in-plane angle $\Delta \alpha =|\alpha _{\textrm{e}}-\alpha _{\textrm{t}}|$ (isotropic case $a_{\textrm{t}}=0$ omitted). Means not sharing uppercase letters differ significantly by the Tukey-test at the $5\%$ significance level. Letters must be compared within the same concentration $a_{\textrm{t}}$.

For the in-plane parameters, we explored all combinations of mean angles $\alpha =0^\circ$, $30^\circ$, $60^\circ$, $90^\circ$ with concentrations $a=0$, 0.5, 1, 2, 5, while the out-of-plane angle $\beta =0^\circ$ and concentration $b=1$ remained fixed. For reasons of symmetry, negative angles are not considered. Errors between the estimated and true parameters are evaluated by the difference for the in-plane concentration and the absolute difference for the mean in-plane angles. The results are summarized in Fig. 5.

The estimate of the true concentration $a_{\textrm{t}}$ seems almost insensitive to the true mean fiber orientation $\alpha _{\textrm{t}}$, except for $a_{\textrm{t}}=5$ where the algorithm underestimates the true concentration with an average error of $-0.745$ (Fig. 5a). In absolute terms, the error increases with increasing $a_{\textrm{t}}$. The opposite behavior can be observed for the estimation error of the mean angle (Fig. 5b). The isotropic case $a_{\textrm{t}}=0$ is not shown because the mean angle in not relevant in this case. Thereby, the precision improves with concentration $a_{\textrm{t}}$ since the uncertainty on the angular peak location reduces for more concentrated dFODs. Errors are independent of the true mean fiber orientation $\alpha _{\textrm{t}}$, show no significant variations (except for three means) and remain small for all values of $a_{\textrm{t}}$ and $\alpha _{\textrm{t}}$, with a maximum of $1.8^{\circ }$.

**Figure 6:**
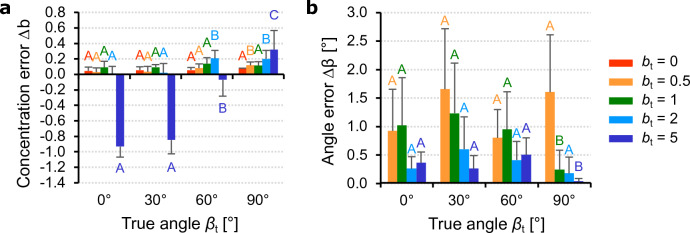
Algorithm precision for out-of-plane parameters. Errors between the estimated (subscript $\textrm{e}$) and true (subscript $\textrm{t}$) out-of-plane parameters (mean and standard deviation of 10 images): (**a**) error in the out-of-plane concentration $\Delta b=b_{\textrm{e}}-b_{\textrm{t}}$; (**b**) error in the out-of-plane angle $\Delta \beta =|\beta _{\textrm{e}}-\beta _{\textrm{t}}|$ (isotropic case $b_{\textrm{t}}=0$ omitted). Means not sharing uppercase letters differ significantly by the Tukey-test at the $5\%$ significance level. Letters must be compared within the same concentration $b_{\textrm{t}}$.

For the out-of-plane precision assessment, we analyzed all combinations of mean angles $\beta =0^\circ$, $30^\circ$, $60^\circ$, $90^\circ$ with concentrations $b=0$, 0.5, 1, 2, 5 while the in-plane angle $\alpha =0^\circ$ and concentration $a=1$ remained fixed. For reasons of symmetry, we have not considered negative angles. The results regarding the errors between the estimated and the true parameters are summarized in Fig. 6.

The estimated out-of-plane concentration parameter (Fig. 6a) shows a moderate dependence on the angle $\beta _{\textrm{t}}$, with low values and no significant differences between $0^{\circ }$ and $60^{\circ }$ for concentrations $b_{\textrm{t}}\le 2$. However, for $b_{\textrm{t}}=5$ the concentration is underestimated at lower angles, but for $\beta _{\textrm{t}}=60^{\circ }$ the error tends to decrease and for $\beta _{\textrm{t}}=90^{\circ }$ it becomes positive. Regarding the mean out-of-plane angle (Fig. 6b), the error shows almost no dependence on $\beta _{\textrm{t}}$, with significant variations between the different concentrations only at $\beta _{\textrm{t}}=90^{\circ }$. Similar to the in-plane case, the errors are generally low with a maximum of $1.65^{\circ }$ and tend to decrease with increasing concentration $b_{\textrm{t}}$.

### Application to skin tomography

We applied the algorithm to compute the dFOD and relative distribution parameters from SHG tomography of human and mouse skin tissue (see Materials and Methods).

#### Human skin

**Figure 7:**
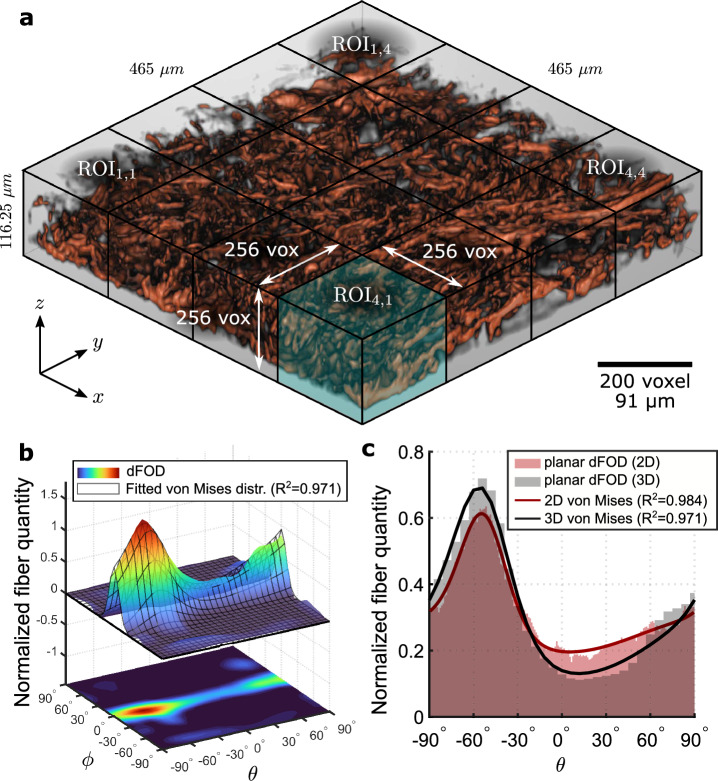
Collagen dFOD (discrete fiber orientation distribution) in human skin: (**a**) SHG (second harmonic generation) tomography of human skin collagen fibers and subdivision of the volume into cubic ROIs; (**b**) 3D surface plot and its projected contour on the $\theta$-$\phi$ plane of the dFOD. The wire-frame plot represents the fitted distribution $\hspace{0.83328pt}\overline{\hspace{-0.83328pt}\rho \hspace{-0.83328pt}}\hspace{0.83328pt}$ using two bivariate von Mises functions ($a_1=2.34$, $b_1=6.15$, $\alpha _1=-54.39^{\circ }$, $\beta _1=-4.33^{\circ }$, $\gamma _1=-8.22^{\circ }$, $\nu _{\textrm{f},1}=0.44$, $a_2=0.38$, $b_2=12.45$, $\alpha _2=90^{\circ }$, $\beta _2=-0.35^{\circ }$, $\gamma _2=0.84^{\circ }$, $\nu _{\textrm{f},2}= 0.56$). (**c**) Comparison of the planar dFOD derived from the 3D discrete Fourier transform and the 2D discrete Fourier transform algorithm ($a_1=3.46$, $\alpha _1=-53.59^{\circ }$, $\nu _{\textrm{f},1}=0.26$, $a_2=0.19$, $\alpha _2=90^{\circ }$, $\nu _{\textrm{f},2}=0.74$).

Figure 7a shows a tomography of human skin collagen fibers constructed from a sequence of SHG images acquired in a volume of $465\,\mu$m $\times$ $465\,\mu$m $\times$ $116.25\,\mu$m (fiber density $\delta = 16.97\%$). A detailed animated view of the tomography is shown in the Supplementary Video 1 available online. In contrast to the artificial straight fibers considered in the previous sections, collagen fibers in the skin are wavy and compacted in bundles of different sizes and diameters^50^. The bundles are in turn interwoven, making it difficult to distinguish them within the volume. However, this does not affect the accuracy of the measured dFOD as the algorithm can detect any fibrous unit within the specified diameters. In addition, wavy fibers can be viewed as a continuous sequence of smaller straight fibers.

The global dFOD, reported in Fig. 7b, is obtained by dividing the volume into 16 cubic ROIs with a size of $M=256$ voxels ($116.25\,\mu$m), as specified in the Materials and Methods section, and summing the respective 16 dFODs. The distribution has an angular resolution of $5.8^{\circ }$ ($D=31$) and is normalized such that the volume under the surface is one. As expected from experimental evidence^51^, the concentration along $\phi$ is higher than along $\theta$, which means that almost all fibers lie in a co-planar plane *x*-*y* with the skin surface. Although the distribution shows only one main peak at around $\theta = -60^{\circ }$ and $\phi = 0^{\circ }$, qualitatively indicating the presence of a single fiber family, a combination of two bivariate von Mises PDFs ($n=2$) (wireframe in Fig. 7b) is adopted to improve the quality of the fit. Note that the algorithm may underestimate the two out-of-plane concentrations $b_i$, because both values are outside the tested range in the out-of-plane precision analyses.

**Figure 8:**
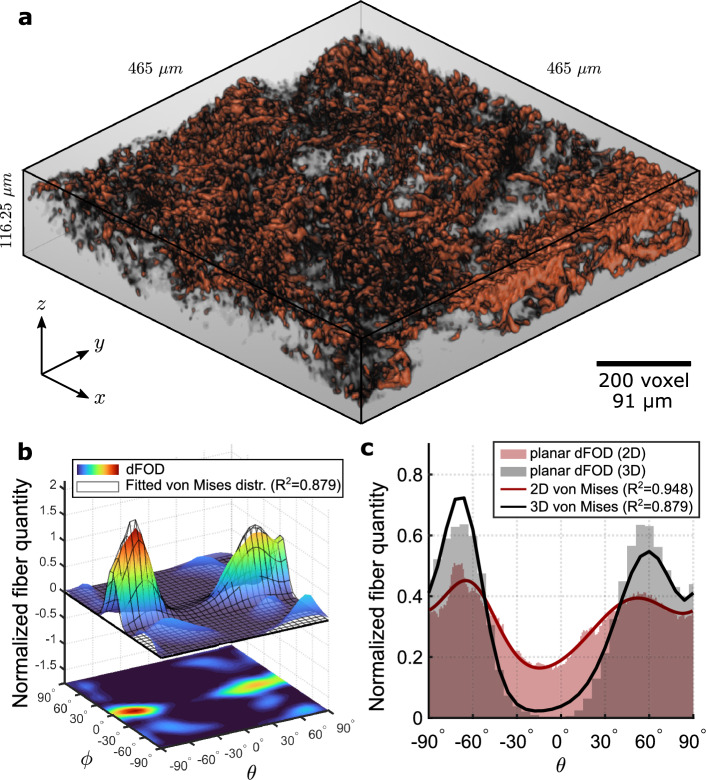
Collagen dFOD (discrete fiber orientation distribution) in mouse skin: (**a**) collagen fiber SHG (second harmonic generation) tomography of mouse skin; (**b**) 3D surface plot and its projected contour on the $\theta$-$\phi$ plane of the dFOD. The wire-frame plot represents the fitted distribution $\hspace{0.83328pt}\overline{\hspace{-0.83328pt}\rho \hspace{-0.83328pt}}\hspace{0.83328pt}$ using two bivariate von Mises functions ($a_1=3.93$, $b_1=11.09$, $\alpha _1=-67.74^{\circ }$, $\beta _1=0.05^{\circ }$, $\gamma _1=-17.73^{\circ }$, $\nu _{\textrm{f},1}=0.44$, $a_2=1.83$, $b_2=9.61$, $\alpha _2=58.85^{\circ }$, $\beta _2=-4.32^{\circ }$, $\gamma _2=7.22^{\circ }$, $\nu _{\textrm{f},2}=0.56$). (**c**) Comparison of the planar dFOD derived from the 3D discrete Fourier transform and the 2D discrete Fourier transform algorithm ($a_1=2.17$, $\alpha _1=-62.05^{\circ }$, $\nu _{\textrm{f},1}=0.29$, $a_2=0.64$, $\alpha _2=50.64^{\circ }$, $\nu _{\textrm{f},2}=0.71$).

In Fig. 7c, the planar distribution computed with the 3D discrete Fourier transform-based algorithm is compared with that obtained using a classic 2D discrete Fourier transform-based approach^32,44,45,52^. With our algorithm (gray histogram), the planar distribution is obtained by summing the dFOD along the direction $\phi$ shown in Fig. 7b, while the 2D case (red histogram) is realized by adding the individual distributions of all in-plane *x*-*y* slices of the tomography, each derived with the 2D algorithm (angular resolution of $1^{\circ }$). The two distributions are qualitatively similar with both peaks located around $\theta =-55^{\circ }$ and showing comparable concentrations. This observation is also confirmed by the parameter set obtained from fitting the 2D von Mises distribution (red solid curve).

#### Mouse skin

Figure 8a shows a tomography of mouse skin collagen fibers constructed from a sequence of SHG images acquired in a volume of $465\,\mu$m $\times$ $465\,\mu$m $\times$ $116.25\,\mu$m (fiber density $\delta = 16.31\%$). Collagen fibers in mouse skin are finer compared to human fibers and warp around hair follicles, appearing as cavities in the tomography. A detailed animated view of the tomography is shown in the Supplementary Video 2 available online.

As for human skin, the global dFOD is calculated by dividing the volume into 16 cubic ROIs with a size of $M=256$ voxels ($116.25\,\mu$m) and summarizing the respective 16 dFODs (schemes of the ROIs are omitted here). The distribution shown in Fig. 8b appears to be more clustered along the horizontal plane *x*-*y* with respect to the human skin, showing two distinct peaks at about $\theta = -65^{\circ }$ and $\theta = 60^{\circ }$ ($\phi = 0^{\circ }$). A combination of two bivariate von Mises PDFs ($n=2$) is adopted for the fitting (wireframe in Fig. 8b).

The comparison between the planar distributions obtained with the 2D and 3D discrete Fourier transform-based algorithms is reported in Fig. 8c. Here the two peaks observed in the distribution constructed from the 3D dFOD in Fig. 8b are also observed in the 2D discrete Fourier transform-based distribution, although the trend seems smoother. In this case, the in-plane parameters present noticeable differences between the two algorithms. This is likely due to the inability of the 2D algorithm to detect fibers with high elevation, where quasi-circular fiber cross-section is detected as isotropicly distributed in the *x*-*y* plane. With the 2D approach, the concentration parameters are lower and the volume fractions appear more unbalanced. In addition, the mean in-plane fiber directions are closer to the *x* direction ($\theta =0^{\circ }$). However, despite the noticeable differences, the two collagen families are well captured by both algorithms.

## Discussion

We proposed a discrete Fourier transform-based algorithm that extends the classical 2D discrete Fourier transform-based methods of measuring fiber orientation to the 3D case^45,49,53,54^. The filter used to identify the orientation of fibers in the 3D space is obtained by rotating a 2D band-pass filter (wedge filter) about its axis. However, in the 3D case, an extra step is required before finding the actual dFOD $d'(\theta ,\phi )$. In particular, a deconvolution process is applied to clean each directional measurement of the interference from all other directions. Such interference is caused by the intersections of the 3D filters pivoting around the fixed frequency point $(u,v,w)=(M/2,M/2,M/2)$ in the frequency domain, where *M* is the size of the 3D cubic image. Unlike the 2D case, where wedge filters can be shaped without overlap, filters in a 3D framework always intersect, even in case they degenerate to discs. Therefore, deconvolution is a necessary step to mathematically obtain a faithful representation of the actual fiber orientation distribution.

The algorithm is based on three parameters that need to be adjusted: the cut-off frequencies $\text{f}_{\rm min}$ and $\text{f}_{\rm max}$ and a spectrum power parameter *q* (see Materials and Methods). The two frequencies are derived directly from the examined maximum and minimum fiber diameters, respectively, while the parameter *q* is calibrated to match the output dFOD with the true distribution of artificial fiber stacks. This parameter behaves similarly to the one proposed by Polzer et al.^45^, with the difference that in their case the entire distribution $d(\theta )$ is raised instead of the spectrum $|{\hat{g}}|$. The three-parameter method gives satisfying results, but more sophisticated filtering techniques developed for 2D algorithms can also be integrated into our method. For example, Witte et al.^46^ proposed an adaptive filtering technique based on the propagation of uncertainties that excludes spectrum magnitudes not carrying fiber-related information, thus, eliminating the need to define specific cut-off frequencies based on fiber diameters.

Given the fundamental importance of *q* in the proposed method, we performed a calibration analysis to assess its influence on the concentration parameters *a* and *b* of the von Mises distribution. Ideally, the errors $\Delta \sigma _{\theta \theta }$ and $\Delta \sigma _{\phi \phi }$ of the measured standard deviations should be independent on the specific dispersion analyzed and dependent only on *q*. In other words, there should be a unique value of *q* for which the errors $\Delta \sigma _{\theta \theta }$ and $\Delta \sigma _{\phi \phi }$ are minimized simultaneously and independently of the true fiber distribution. In practice, however, the errors also depend on the specific fiber dispersion considered. From Fig. 2b,c we can deduce that for increasing concentrations the algorithm tends to produce a smoother dFOD than the true one. This suggests that a larger spectrum power parameter *q* might be necessary to increase the sharpness of the measured distribution.

This behavior is also reflected in the Figs. 5a and 6a, in which the true concentrations are underestimated for high concentrations ($a_{\textrm{t}} = 5$, $b_{\textrm{t}}= 5$). Their precision would improve if the value of *q* were increased, but on the other hand the algorithm would overestimate at lower concentrations. Since our goal was to get accurate estimates of the fiber dispersion parameters in most of the cases, we identified $q=2.4$ as an acceptable compromise. In contrast to the concentration parameters, the true mean orientation angles $\alpha _{\rm t}$ and $\beta _{\rm t}$ depend only on the distribution peaks and are not directly affected by *q*. As shown in Figs. 5b and 6b, their precision generally increases with concentration as the uncertainty about the peak location is reduced for sharper distributions. Note that the precision of the parameters depends on the algorithm, while the nonlinear least squares fit ensures a reliable estimate of the dispersion parameters. The robustness analysis revealed that the reliability of our method is not affected by the number of fibers *N* and their diameter *t*, although they limiting factors may become larger in the out-of-plane measurements when fiber density $\delta$ is greater than about $25\%$. In order to understand the capabilities of the proposed method in detail, future analyses should deal with, e.g., the influence of noise^46^ and the influence of the fiber waviness.

Despite the approximations and limitations discussed, the algorithm provides accurate quantitative descriptions of the spatial arrangement of complex fiber dispersions. In contrast to 2D approaches, where fibers that are strongly inclined from the *x*-*y* plane are difficult to detect due to their small cross-section, our method can consider all fibers with any orientation in 3D space. Differently from existing 3D methods^13,47^, which provide separate distributions along $\theta$ and $\phi$, our algorithm provides a complete description of the spatial fiber distribution over the entire 3D angular domain. This allows to identify the different fiber families in the volume and to compute for each family the specific set of parameters without ambiguities. In fact, when multiple fiber families are present, the different peaks cannot be combined together if only the separate in-plane and out-of-plane distributions are known, without information on how they co-vary in the two-dimensional angular domain.

In practice, as shown for human and mouse skin, this scenario rarely occurs in biological soft tissues where fibers are mainly aligned in the *x*-*y* plane (Figs. 7b and 8b). In these cases, the fiber families and their in-plane parameters can be computed from the in-plane distribution, while the out-of-plane parameters can be extracted from the 2D distribution of a vertical tissue section. However, this is still an approximation since all the fiber families are assigned a unique concentration *b*^23^. Another benefit of full 3D distributions is the ability to measure the rolling angle $\gamma$, which allows for general rotations of the fiber family in space and compensates for slight planar misalignments of the samples during 3D imaging.

In addition, our method is fast and robust. To analyze stacks of $1024 \times 1024 \times 256$ voxels ($465\,\mu$m$\times$ $465\,\mu$m $\times$ $116.25\,\mu$m) the overall computation took 65 s for human skin (60.1 s for the computation of the raw dFOD and 4.9 s for the deconvolution), and 61 s for mouse skin (56.2 s for the computation of the raw dFOD and 5.1 s for the deconvolution, on a 2.20GHz CPU, 32GB RAM Desktop PC). Of course, the computation speed depends on the resolution of the dFOD, since the number of computations increases proportionally with the angular interval $D^2$. Also, memory can be a limiting factor, since the free memory needed to store the filters increases proportionally to $D^2\times M^3$. However, with a value of $D=31$ adopted in the provided examples, distributions with a good angular resolution can be obtained in a reasonable computation time.

The robustness analysis showed that the algorithm reliably predicts the same FOD for stacks with a fiber density of up to $25\%$. This represents a major advantage over pixel-by-pixel methods, which are affected by orientation uncertainties in the pixels when two or more fibers overlap in very dense fiber dispersions^43^. In general, approaches based on discrete Fourier transforms are less affected by image complexity since fiber orientations are computed from the frequency domain, which is insensitive to fiber overlaps in the spatial domain^32^. It is important to note that the proposed method requires high-quality stacks with a *z* resolution comparable to that in the *x*-*y* plane, which are typically expensive and time-consuming to obtain. The SHG stack of human and mouse skin collagen in Figs. 7a and 8a (shown after the vertical resampling) is acquired with our equipment at the highest possible *z* resolution ($\Delta z = 0.57\,\mu$m), each requiring up to 2h to complete the acquisition. In addition, to reduce the blurring effect along the *z* direction due to the diffraction of the confocal microscope, we pre-processed the tomographies by applying three-dimensional deconvolution. This process is slow and computationally expensive, but recent deep learning techniques have shown promising results in deconvolution of microscope images^55^ as well as in reducing noise^56^. Furthermore, since the algorithm is based on grayscale 3D images, any technique can be used to capture the stacks, making our method applicable to various imaging technologies, including x-ray computational tomography (xCT)^57^, optical coherence tomography (OCT), and ultrasound imaging^58,59^.

In summary, the presented novel algorithm is able to reliably quantify the fiber network obtained from 3D images by determining the dispersion parameters of each individual fiber family. These parameters can be used to inform mechanical models of soft fiber-reinforced materials and biological tissues that account for non-symmetrical fiber dispersion.

## Materials and methods

### Orientation and distribution of fibers

**Figure 9:**
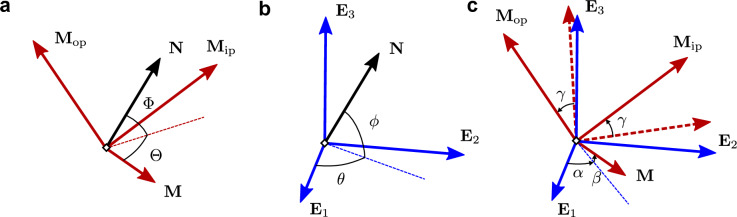
Schematic representation of the orientation of the unit fiber ${\textbf{N}}$ (in black), the global basis $\{{\textbf{E}}_a\}_{a=1,2,3}$ (in blue) and the principal basis $\{{\textbf{M}}$, ${\textbf{M}}_{\textrm{ip}}$, ${\textbf{M}}_{\textrm{op}}\}$ (in red): (**a**) unit vector ${\textbf{N}}$ in the principal frame; (**b**) unit vector ${\textbf{N}}$ in the global basis; (**c**) rotation of the principal basis with respect to the global basis using the Tait-Bryan angles. Dashed vectors represent ${\textbf{M}}_{\textrm{ip}}$ and ${\textbf{M}}_{\textrm{op}}$ before the rotation about ${\textbf{M}}$.

A general fiber orientation distribution can be assumed as a combination of a finite number of fiber families, each of which is described by a PDF $\rho ({\textbf{N}})$ providing the normalized angular density of the fibers in the direction of the unit vector ${\textbf{N}}$. We introduce two different bases: a general basis $\{{\textbf{E}}_a\}_{a=1,2,3}$, and a principal basis of the fibers $\{{\textbf{M}}$, ${\textbf{M}}_{\textrm{ip}}$, ${\textbf{M}}_{\textrm{op}}\}$, where ${\textbf{M}}$ represents the mean fiber direction, $({\textbf{M}},{\textbf{M}}_{\textrm{ip}})$ is the plane containing the mean direction of the fiber family and ${\textbf{M}}_{\textrm{op}}$ is the out-of-plane normal (Fig. 9). With respect to the principal frame, the fiber unit vector ${\textbf{N}}$ is written as

1 $$\begin{aligned} {\textbf{N}}(\Theta ,\Phi )=\cos (\Phi )\cos (\Theta ){\textbf{M}}+\cos (\Phi )\sin (\Theta ) {\textbf{M}}_{\textrm{ip}}+\sin (\Phi ){\textbf{M}}_{\textrm{op}}\,, \end{aligned}$$

where $\Theta$ and $\Phi$ are the azimuthal and elevation angles of the fiber unit vector ${\textbf{N}}$ with respect to the principal frame (Fig. 9a).

The PDF can be decomposed as a bivariate distribution of the form $\rho ({\textbf{N}})=\rho (\Theta ,\Phi )=\rho _{\textrm{ip}}(\Theta )\rho _{\textrm{op}}(\Phi )$, where $\rho _{\textrm{ip}}$ and $\rho _{\textrm{op}}$ represent the in-plane and out-of-plane distribution functions, respectively. In terms of the global frame, the general expression of the PDF is as follows

2 $$\begin{aligned} \rho ({\textbf{N}})=\rho (\theta ,\phi )=\rho _{\textrm{ip}} (\Theta (\theta ,\phi ))\rho _{\textrm{op}}(\Phi (\theta ,\phi )), \end{aligned}$$

where $\theta$ and $\phi$ represent the azimuthal and elevation angles of the unit vector ${\textbf{N}}(\theta ,\phi )$ in the global frame (Fig. 9b).

We consider a rigid rotation of the fiber families in Euclidean space by introducing the Tait-Bryan angles $\alpha$, $\beta$, $\gamma$. The first two angles denote the azimuthal and elevation angles of the mean direction ${\textbf{M}}$ and $\gamma$ the rotation of the principal fiber frame by ${\textbf{M}}$ (Fig. 9c). In general, the functions $\Theta (\theta ,\phi )$ and $\Phi (\theta ,\phi )$ in Eq. (2) are implicit and depend on the three angles $\alpha$, $\beta$, $\gamma$. Explicit expressions are only available for some simple cases, such as $\beta =\gamma =0$ for which $\Theta =\theta -\alpha$ and $\Phi =\phi$.

The functions $\rho _{\textrm{ip}}(\Theta )$ and $\rho _{\textrm{op}}(\Phi )$ are provided by two $\pi$-periodic von Mises distributions of the form^23^

3 $$\begin{aligned} \rho _{\textrm{ip}}(\Theta )=\dfrac{\exp (a\cos 2\Theta )}{I_0(a)}, \qquad \rho _{\textrm{op}}(\Phi )=2\sqrt{\dfrac{2b}{\pi }}\dfrac{\exp [b(\cos 2\Phi -1)]}{{{\,\textrm{erf}\,}}(\sqrt{2b})}, \end{aligned}$$

where *a* and *b* are the in-plane and out-of-plane concentration parameters, respectively, $I_0(x)=\frac{1}{\pi }\int _{0}^{\pi }\exp (x\cos t)\textrm{d}t$ is the modified Bessel function of the first kind of order zero, and ${{\,\textrm{erf}\,}}$ is the error function given by ${{\,\textrm{erf}\,}}(x)=\frac{2}{\sqrt{\pi }}\int _{0}^{x}\exp (-t^2)\textrm{d}t$. Large values of *a* and *b* correspond to a fiber distribution with a high degree of alignment along the mean fiber direction ${\textbf{M}}$, while for $a,b\rightarrow 0$ an isotropic distribution is obtained. This choice for the in-plane and out-of-plane functions satisfies the symmetry requirement $\rho ({\textbf{N}})=\rho (-{\textbf{N}})$, or equivalently $\rho (\theta ,\phi )=\rho (\theta +180^\circ ,-\phi )$, and the normalization condition over the unit sphere ${\mathbb {S}}^2$, $\frac{1}{4 \pi }\int _{{\mathbb {S}}^2}\rho ({\textbf{N}})\textrm{d}\Omega =1$, required in mechanical models^23,24^.

### 3D discrete Fourier transform-based algorithm for measuring fiber orientation distributions

We have developed a novel method based on the 3D discrete Fourier transform in combination with a so-called funnel filtering to extract directional data from three-dimensional images. The algorithm is implemented in a custom Matlab code.

#### Raw discrete fiber orientation distribution

**Figure 10:**
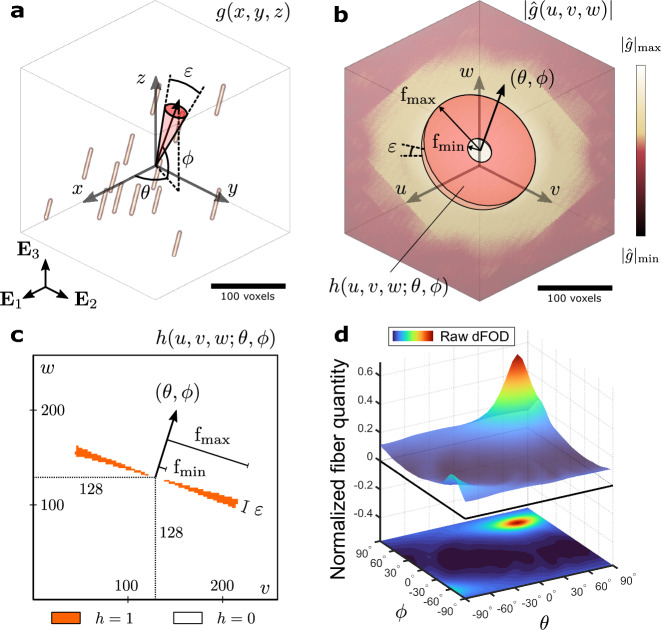
Illustration of the 3D discrete Fourier transform algorithm: (**a**) signal *g*(*x*, *y*, *z*) of a $256 \times 256 \times 256$ representative image in the discrete spatial domain with $N=14$ fibers aligned to $(\theta ,\phi )=(60^{\circ },60^{\circ })$; (**b**) center-shifted spectrum $|{\hat{g}}(u,v,w)|$ in the frequency domain, with a sketch of the overlapping funnel filter sampling the frequencies associated with the fibers with orientations within the cone, shown in (**a**), with mean direction $(\theta ,\phi )=(60^{\circ },60^{\circ })$ and opening $\varepsilon$; (**c**) cross section of the filter in the local *v*-*w* plane at $u=128$. The filter is axisymmetric about the direction $(\theta ,\phi )$ (note that the symmetry axis shown is slightly skewed with respect to the *v*-*w* plane); (**d**) raw dFOD (discrete fiber orientation distribution) of the representative fiber dispersion shown in (**a**) using $q=2.4$, and $D=31$, corresponding to an angular resolution of $\Delta \theta =\Delta \phi = 5.8^{\circ }$ ($\varepsilon$ is taken to be equal to $\Delta \theta =\Delta \phi$).

Let *g*(*x*, *y*, *z*), $g:{\mathbb {N}}^3\rightarrow {\mathbb {R}}$, be the signal in the discrete spatial domain representing a $M\times M\times M$ image (voxel volume). We assume that the *x*, *y*, *z* axes are aligned with the global basis vectors ${\textbf{E}}_1, {\textbf{E}}_2, {\textbf{E}}_3$, so that the definition of the azimuthal and elevation angles $\theta$ and $\phi$ is the same in both frames. If *g*(*x*, *y*, *z*) depicts a bundle of straight fibers all oriented along a certain direction $(\theta ,\phi )$ (Fig. 10a), then its spectrum ${\hat{g}}(u,v,w)$, ${\hat{g}}:{\mathbb {N}}^3\rightarrow {\mathbb {C}}$, is defined by the 3D discrete Fourier transform

4 $$\begin{aligned} {\hat{g}}(u,v,w) = \sum _{x=0}^{M-1}\sum _{y=0}^{M-1}\sum _{z=0}^{M-1}g(x,y,z)e^{-i2\pi \left( \frac{ux}{M}+\frac{vy}{M}+\frac{wz}{M}\right) }, \end{aligned}$$

where the dominant magnitudes $|{\hat{g}}(u,v,w)|$ are scattered in a plane that pass through $(u,v,w)=(M/2,M/2,M/2)$ and perpendicular to the frequency direction $(\theta ,\phi )$ (yellow voxels in Fig. 10b). Note that the values of the spectrum are shifted such that the lower frequencies occupy the central position, with frequency $\textrm{f}=0$ located at $(u,v,w) = (M/2,M/2,M/2)$. Gibbs artifacts characterized by high magnitudes distributed along the *u*, *v*, *w* axes are removed by tapering *g*(*x*, *y*, *z*) with a 3D Tukey window with $40\%$ cosine before the transform.

Note that the orientation of the plane in the frequency domain is invariant with changes in fiber position in the spatial domain. Therefore, the sum of the moduli $|{\hat{g}}(u,v,w)|$ filtered from this plane is a measure of the amount of straight fibers oriented towards $(\theta ,\phi )$. Due to the aliasing filter plane, however, this measure reacts sensitively to small variations in the sampled direction $(\theta ,\phi )$. To reduce this sensitivity, we perform the computation over a finite solid angle rather than the exact direction, so the filter corresponds to a volume obtained by enveloping all planes of all directions within the solid angle. The volume is still aliased along its boundary (Fig. 10c), but it represents only a lower fraction of the total number of frequencies filtered. Therefore, it is less sensitive to small changes in the sampled direction. To exclude the part of the spectrum that does not contain relevant fiber information the filter is reduced to include magnitudes only between minimum and maximum cut-off frequencies $\textrm{f}_{\rm min}$ and $\textrm{f}_{\rm max}$. Assuming a conical solid angle with the amplitude $\varepsilon$ and the mean direction $(\theta ,\phi )$ the funnel-shaped filter is defined by

5 $$\begin{aligned} h\left( u,v,w;\theta ,\phi \right) = {\left\{ \begin{array}{ll} 1 &{} {\text{if}}\;\;{\tilde{u}}^2-\tan \left( \varepsilon /2\right) ^2\left( {\tilde{v}}^2+{\tilde{w}}^2\right) \le 0\;\; {\text{and}} \;\; {\textrm{f}}_{\rm min}\le \sqrt{{\tilde{u}}^2+{\tilde{v}}^2+{\tilde{w}}^2}\le {\textrm{f}}_{\rm max},\\ 0 &{} \text {else,} \end{array}\right. } \end{aligned}$$

where $\llbracket {\tilde{u}},{\tilde{v}},{\tilde{w}}\rrbracket ^{\mathrm T}= {\textbf{R}}_w^{-1}(\theta ) {\textbf{R}}_v^{-1}(-\phi )\llbracket u-M/2,v-M/2,w-M/2\rrbracket ^{\mathrm T}$, ${\textbf{R}}_w$ and ${\textbf{R}}_v$ are the rotation matrices around the *w* and *v* axes, respectively (Fig. 10b).

We can then define the raw dFOD as a function $d:{\mathscr {O}}\rightarrow {\mathbb {R}}$,

6 $$\begin{aligned} d(\theta ,\phi )=\sum _{u=0}^{M-1}\sum _{v=0}^{M-1}\sum _{w=0}^{M-1}|{\hat{g}}(u,v,w)|^q h(u,v,w;\theta ,\phi ), \end{aligned}$$

computed along all the discrete directions $(\theta ,\phi )\in {\mathscr {O}}=\left\{ \left( -90^\circ +\frac{2i-1}{2}\Delta \theta ,-90^\circ +\frac{2j-1}{2}\Delta \phi \right) | i,j=1,\ldots ,D\right\}$, where $\Delta \theta =\Delta \phi =180^\circ /D$ defines the angular resolution in terms of *D* evenly spaced angles (Fig. 10d).

In Eq. (6) we introduced an exponent *q* to correct the distribution. Usually, in 2D discrete Fourier transform-based algorithms^53,54,60^, the exponent is set to $q=2$, but other values can be used to achieve better results^45^. The calibration analysis is used to identify the optimal value of the parameter to match the dFOD to the prescribed distribution of artificial fiber images. To ensure a thorough measurement of all fibers in the discrete angle set, we assume that the cone opening corresponds to the angular resolution $\varepsilon =\Delta \theta =\Delta \phi$. Assuming an image size of $M=256$ voxels, which is suitable for most biological applications, and without assumptions about the investigated fiber diameter, we set the cut-off frequencies to $\text{f}_{\rm min}=4$ and $\textrm{f}_{\rm max}=43$ to exclude high frequencies associated with noise and low frequencies associated with exposition variations within the image^54^. Given the relationship $\textrm{f}=M/(2t)$, this frequency range corresponds to fiber diameters between $t=32$ and $t=3$ voxels. However, this does not necessarily mean that fibers larger than 32 voxels and smaller than 3 voxels will not be detected, since the information conveyed by the discrete Fourier transform about a specific fiber diameter $t'$ is spread in a band of about $\textrm{f}'\pm 0.1\textrm{f}'$^53^.

An essential aspect of the proposed method is that the signal *g* and accordingly the spectrum ${\hat{g}}$ correspond to cubes with the same size $M \times M \times M$. Different sizes along the three dimensions of *g* and ${\hat{g}}$ would result in an inaccurate dFOD since the higher number of frequencies along the dominant direction would make a larger contribution with respect to other directions in the Eq. (6).

Another aspect to consider is computational efficiency. For computational reasons, all filters are computed before the analysis, which requires free memory proportional to the product between the number of directions $D^2$ and the number of voxels in the filter $M^3$. This might become too demanding when analyzing high-resolution 3D images ($M\approx 1024$). To limit the amount of memory required for the computations while maintaining a cubic shape for ${\hat{g}}$, the 3D image is divided into ROIs of $256 \times 256 \times 256$ voxels. Then the raw dFOD $d(\theta ,\phi )$ is computed as the sum of the raw dFODs of each individual ROI. The sum does not require any weighting, since ${\hat{g}}$ already takes into account the luminance, i.e. the overall amount of fibers within the ROI. If the dimensions are not a multiple of 256, the 3D image can be divided into cubic ROIs of different size (possibly close to 256), which can be scaled up or down to $M=256$ by volumetric interpolation before the 3D discrete Fourier transform.

#### Deconvolution of the dFOD

The distribution $d(\theta ,\phi )$ needs to be deconvoluted to obtain the actual dFOD $d'(\theta ,\phi )$. Because the filters overlap in the frequency domain, the measured amount of fibers along a generic direction $I=(\theta _i,\phi _j)$ contains a percentage of the actual amount of fibers from all other directions $J=(\theta _k,\phi _l)$ that is proportional to the number of shared magnitudes between filters along *I* and *J* directions.

Mathematically, we can write the raw dFOD in vector form by the linear relationship ${\textbf{d}} = {\textbf{K}} {\textbf{d}}'$, where ${\textbf{d}}'$ denotes the unknown deconvoluted dFOD in vector form. The symmetric convolution kernel matrix $K_{IJ}$ represents the normalized number of common magnitudes between filters *I* and *J*. Since the distributions can be arbitrarily rearranged from matrix to vector, we use the index rule $I=(j-1)D+i$. We obtain the deconvoluted vector ${\textbf{d}}'$ using the iteratively constrained Tikhonov-Miller algorithm, which gives the optimal non-negative solution to

7 $$\begin{aligned} {{\,\mathrm{arg\ min}\,}}_{{\textbf{d}}'}\left( \Vert {\textbf{K}}{\textbf{d}}'-{\textbf{d}}\Vert ^2 +\lambda \Vert {\textbf{L}}{\textbf{d}}'\Vert _{2}^2\right) . \end{aligned}$$

Here the second term depends on the discrete Jacobian operator matrix ${\textbf{L}}$ and the regularization parameter $\lambda \in \left[ 0,1\right]$ which was introduced to avoid noise amplification in the solution^61^. This method is required because direct matrix inversion would provide noisy and possibly non-physical (negative values) results. A step-size of 0.1 and $\lambda =10^{-3}$ is used for the iterations. Once the solution is found, the deconvoluted vector ${\textbf{d}}'$ is rearranged again as a matrix using the aforementioned reordering rule, providing the sought dFOD $d'(\theta ,\phi )$.

### Fiber orientation distribution parameters

To estimate the parameters of the fiber orientation distribution, the function $\hspace{0.83328pt}\overline{\hspace{-0.83328pt}\rho \hspace{-0.83328pt}}\hspace{0.83328pt}(\theta ,\phi )=\rho (\theta ,\phi )\cos (\phi )$ is fitted to the deconvoluted dFOD $d'(\theta ,\phi )$, where $\rho (\theta ,\phi )$ is defined in Eqs. (2) and (3). The cosine of the elevation angle $\phi$ accounts for the surface area $\textrm{d}\Omega$ on the unit sphere ($\textrm{d}\Omega =\cos {\phi }\textrm{d}\theta \textrm{d}\phi$). Note that $d'(\theta ,\phi )$ represents the normalized quantity of fibers along each discrete direction, while the PDF $\rho (\theta ,\phi )_n$ needs to be multiplied by the surface element area to obtain a normalized fiber quantity. For the multi-fiber distribution, a linear combination $\hspace{0.83328pt}\overline{\hspace{-0.83328pt}\rho \hspace{-0.83328pt}}\hspace{0.83328pt}=\sum _n \nu _{\textrm{f,}n}\hspace{0.83328pt}\overline{\hspace{-0.83328pt}\rho \hspace{-0.83328pt}}\hspace{0.83328pt}_n$ is used, where $\hspace{0.83328pt}\overline{\hspace{-0.83328pt}\rho \hspace{-0.83328pt}}\hspace{0.83328pt}_n$ is the function with respect to the *n*-th fiber family and $\nu _{\textrm{f,}n}$ its volume fraction, with $\sum _n \nu _{\textrm{f,}n}=1$.

The operation is performed in Matlab using the integrated nonlinear least squares function lsqnonlin. To assess the quality of the fit, we use the coefficient of determination $R^2$.

### Statistical analysis

A two-way ANOVA analysis is used to calibrate the algorithm and to analyze the precision of the estimated parameters of the in-plane and out-of-plane fiber orientation distribution. Instead, a one-way ANOVA is used to evaluate the robustness of the algorithm. For all analyses, a post-hoc Tukey HSD (honestly significant difference) test is used to assess pairwise differences between groups. The results are considered significant at the $5\%$ level. Due to the high number of pairwise comparisons, we use the compact letter display with uppercase letters to report the results. The data analysis is carried out employing the Real Statistics Resource Pack software^62^.

### Artificial fiber dispersion volume

Using a custom Matlab code, we generated artificial grayscale 3D images with a number *N* of straight fibers with known fiber orientation distribution within a volume of $M\times M\times M$ voxels. The midpoint $(x,y,z)_k$ of each fiber, $k=1,\dots ,N$, is chosen randomly according to a three-dimensional uniform distribution, while the orientation angles $(\theta ,\phi )_k$ are sampled from the PDF given in Eq. (2) using a rejection method. The sampling is performed by generating uniformly distributed random points within the three-dimensional domain ${\mathcal {D}}=\left\{ (\theta ,\phi ,r)|-90^\circ \le \theta \le 90^\circ ,-90^\circ \le \phi \le 90^\circ ,0\le r \le \max \left( \hspace{0.83328pt}\overline{\hspace{-0.83328pt}\rho \hspace{-0.83328pt}}\hspace{0.83328pt}\right) \right\}$ and selecting only those points that fall under the function $\hspace{0.83328pt}\overline{\hspace{-0.83328pt}\rho \hspace{-0.83328pt}}\hspace{0.83328pt}(\theta ,\phi )$.

Once the fiber positions $(x,y,z)_k$ and orientations $(\theta ,\phi )_k$ are computed, a 3D binary image of $M\times M\times M$ voxels is generated with an intensity $Y=1$ for voxels belonging to a fiber and $Y=0$ elsewhere. All fibers are of the same aspect ratio of more than 10, in order to enable a meaningful evaluation of the FOD using the discrete Fourier transform methods^53,54^. The image is then smoothed using a Gaussian kernel with a standard deviation of 0.7 voxel to reduce the sharpness of fibers that might introduce artifacts in the spectrum. This operation converts the image from binary to the grayscale image. Since smoothing reduces the maximum intensity $Y_{\rm max}$, all voxels are scaled by a factor $Y_{\textrm{max}}^{-1}$ to restore the maximum intensity to 1.

### Collagen fiber tomography

Images of collagen fibers were acquired from human skin samples harvested from the abdominal region during a routine surgical procedure. The study was approved by the Regional Committee for Medical and Health Research Ethics (Project ID: 474249). All examinations were performed according to the rules for the investigation of human subjects set out in the Declaration of Helsinki. All study participants provided written informed consent.

In addition, we analyzed *ex vivo* skin samples from the dorsal region of mice. The skin tissue was obtained from mice used for *in vivo* studies in another project. After sacrifice by cervical dislocation under isoflurane anesthesia performed manually by trained personnel, the tissue was donated to the present project for *ex vivo* studies. The animal laboratory (Department of Comparative Medicine at Norwegian University of Science and Technology) is approved for animal tests by the Norwegian Food and Animal Safety Authority in document VSID 3506. In this document the laboratory’s systems for animal care and ethics are approved according to the relevant national and EU regulations.

Samples were cleaned from adipose tissue and stored at $-28^\circ$ within 2h of harvest. Prior to SHG imaging, tissue was thawed at room temperature ($22^\circ$) and prepared according to the SeeDBp protocol^63^, consisting of a fixation step in $4\%$ paraformaldehyde for 12h and 6 optical clearing steps in fructose solution in $0.1\times$PBS of increasing concentration. Specifically, the samples were incubated for 4h each in a $20\%$, $40\%$, $60\%$ w/v solution, then for 12h each in a $80\%$, $100\%$ w/v solution and finally for 24h in a $80.2\%$ w/w solution (SeeDB solution). All steps took place at $25^\circ$C. This technique allows biological tissues to be cleared without significant morphological changes, even in the case of fibrous tissue^64^. After completing the clearing process, the samples were placed in a press-to-seal silicone isolator (CoverWell™ Imaging Chambers, Grace BIO-Labs, Oregon, USA) filled with SeeDBp solution and sealed with two rectangular glass coverslips at the top and bottom.

For SHG imaging of collagen fibers, we used the confocal multiphoton microscope Leica TCS SP8 (Leica Microsystem, Germany) with a Leica HCX IRAPO $25\times$, NA 0.95 water objective with a working distance of 2.4 mm. The second harmonic of collagen is induced using a multiphoton laser source tuned at 890 nm (Chameleon Ultra I; Coherent Corp., Saxonburg, PA, United States) and the signal emitted at 445 nm is detected in the forward and backward directions. The images were acquired on a square target of $465\,\mu$m $\times$ $465\,\mu$m with a *x*-*y* resolution of $0.454\,\mu$m/px every $0.57\,\mu$m (minimum vertical step of the microscope) in the *z* direction, for a total of $\sim 116.25\,\mu$m scanned thickness. To compensate for blurring effects due to light diffraction from the confocal microscope, especially along the *z* direction, the 3D image was pre-processed in Fiji^65^ using the open source DeconvolutionLab2 plugin for deconvolution microscopy^61^. In particular, we used the Richardson-Lucy algorithm with total-variation regularization (regularization parameter $\lambda =10^{-3}$, 30 iterations), adopting the Gibson and Lanny 3D optical model for the point spread function^66,67^. Then, to match the *z* spacing with the *x*-*y* resolution, the 3D image was resampled vertically using the imresize3 Matlab function with a stretch factor of $\Delta z/\Delta x = 0.57/0.454\approx 1.26$.

### Supplementary Information

Supplementary Video 1.

Supplementary Video 2.

## Data Availability

The data that supports the results within this paper are available from the corresponding author upon reasonable request.

## Code availability

The custom code of the proposed method is available in the Zenodo repository at https://zenodo.org/doi/10.5281/zenodo.10119628.
